# On doing multi-act arithmetic: A multitrait-multimethod approach of performance dimensions in integrated multitasking

**DOI:** 10.3389/fpsyg.2022.946626

**Published:** 2022-08-18

**Authors:** Frank Schumann, Michael B. Steinborn, Hagen C. Flehmig, Jens Kürten, Robert Langner, Lynn Huestegge

**Affiliations:** ^1^Mittweida University of Applied Sciences, Mittweida, Germany; ^2^Julius-Maximilians-Universität Würzburg, Würzburg, Germany; ^3^Technische Universität Dresden, Dresden, Germany; ^4^Medical Faculty, Institute of Systems Neuroscience, Heinrich Heine University Düsseldorf, Düsseldorf, Germany; ^5^Institute of Neuroscience and Medicine (INM-7: Brain and Behaviour), Research Center Jülich, Jülich, Germany

**Keywords:** sustained attention, concentration, vigilance, reliability, cognitive control

## Abstract

Here we present a systematic plan to the experimental study of test–retest reliability in the multitasking domain, adopting the *multitrait-multimethod (MTMM) approach* to evaluate the psychometric properties of performance in *Düker-type* speeded multiple-act mental arithmetic. These form of tasks capacitate the experimental analysis of *integrated multi-step processing* by combining multiple mental operations in flexible ways in the service of the overarching goal of completing the task. A particular focus was on scoring methodology, particularly measures of response speed variability. To this end, we present data of two experiments with regard to (a) test–retest reliability, (b) between-measures correlational structure, (c) and stability (test–retest practice effects). Finally, we compared participants with high versus low performance variability to assess ability-related differences in measurement precision (typically used as proxy to “simulate” patient populations), which is especially relevant in the applied fields of clinical neuropsychology. The participants performed two classic integrated multi-act arithmetic tasks, combining addition and verification (Exp. 1) and addition and comparison (Exp. 2). The results revealed excellent test–retest reliability for the standard and the variability measures. The analysis of between-measures correlational structure revealed the typical pattern of convergent and discriminant relationships, and also, that absolute response speed variability was highly correlated with average speed (*r* > 0.85), indicating that these measures mainly deliver redundant information. In contrast, speed-adjusted (relativized) variability revealed discriminant validity being correlated to a much lesser degree with average speed, indicating that this measure delivers additional information not already provided by the speed measure. Furthermore, speed-adjusted variability was virtually unaffected by test–retest practice, which makes this measure interesting in situations with repeated testing.

## 1. Introduction

Sustaining mental focus to continuous activity is perceived as arduous and often hard to keep up over a prolonged time period (Humphreys and Revelle, 1984; Langner and Eickhoff, 2013). This is particularly true when a task requires more than a single mental operation and instead consists of a composite of subordinate actions, or a conglomerate of nested mental acts which form a coordinated ensemble where the whole is more than the integrated sum of its parts (Greenwald, 1970, 1972; Navon and Gopher, 1979; Vandierendonck et al., 2010; Huestegge et al., 2014). Everyday examples can be found in the borrow operation in complex subtractions, or the carry operation in complex multiplications (Ashcraft, 1992). Researchers and practitioners in work-related diagnostic settings often employ speeded (elementary and multitasking) tests for the purpose of assessing individuals’ abilities in the context of personnel selection and classification (Cronbach and Meehl, 1955; Campbell and Fiske, 1959). While elementary forms are typically constructed as “*Bourdon-style”* tests, putting excessive demands on perceptual identification, more complex (multitasking) forms typically utilize primary cultural techniques such as mental arithmetic, tapping more into the supervisory scheduling of multiple operations in close temporal succession, which, in their entirety, form an integrated holistic *“Düker-style”* test (Düker, 1949). The present study presents a psychometric analysis of tasks from the latter (multi-operation) category adopting the multitrait-multimethod (MTMM) approach (Campbell and Fiske, 1959; Miller and Ulrich, 2013).

### 1.1. The psychometrics of multitasking

In their momentous work that formed a milestone in the development of psychometric theory, Düker and Lienert (1949) stated that “…*Kapazität entsteht durch das geordnete Zusammenwirken von Einzeltätigkeiten zu einer Handlung durch optimale Koordination*…*”. [“*…*capacity is created by the well-ordered interplay of individual operations to form an action through optimized coordination*…*”].* The authors held the position that the concept of general cognitive ability is best represented by a test that requires the speeded coordination of elemental mental acts (e.g., recording, calculating, memorizing, rule retrieval, ordering and sequencing, etc.) serving the overarching objective to complete the task. Based on this theorizing, Düker and Lienert (1949) developed the Konzentrations-Leistungs-Test (KLT) (“concentration ability test”), which later on became one of the standard measures in the psychometric testing of elementary cognitive ability. In contrast to *Bourdon-type* cancelation tests (Bates and Lemay, 2004), the KLT requires individuals to work on relatively complex *compound multiple-act* arithmetic tasks over a period of about 20 min. A pivotal point is the inherent multitasking nature of the task (Logan, 1979; Ashcraft, 1992; Pashler, 1994b; Bruning and Manzey, 2018), with each item requiring multistep mental operations that include elemental acts (e.g., addition, subtraction, memorizing) serving to complete the overall task as the primary goal (Oberauer, 2002, 2003; Botvinick and Bylsma, 2005a,b; Janczyk and Kunde, 2010, 2020; Herbort and Rosenbaum, 2014).

Although Düker and Lienert (1949) were less concerned with the cognitive analysis of tasks but with the utility of cognitive operations for psychometric testing, they are regarded as pioneers in the history of a cognitive–psychometric discipline (Pieters, 1983, 1985; Blotenberg and Schmidt-Atzert, 2019a,b). Unlike most psychometricians at this time, who considered test reliability merely a statistical issue to be resolved by simple score accumulation methods (cf. Cronbach, 1947; Lord and Novick, 1968; Kahneman et al., 2021, pp. 55–69), the authors went even further, asking of what exactly determines test reliability and how these factors can be modeled in an experimental setting. According to their view, a psychologically substantiated perspective of reliability must offer the possibility to theorize on the underlying processes that either promote or hamper measurement precision (e.g., reliability affected by motivation, task complexity, repeated testing, etc.). For example, to understand what is meant by task complexity, researchers typically distinguish between automatic (intuitive) and controlled (reflective) components of arithmetic processing (Ashcraft and Battaglia, 1978; Manstead and Semin, 1980; Strack and Deutsch, 2004), and a standard procedure to measure these components is to experimentally manipulate item difficulty in some way, for example, by varying arithmetic-chain length (Steinborn and Huestegge, 2016, 2017, 2020), adding a memory load (Logan, 1979; Gopher, 1996; Vallesi et al., 2014), or by dynamically switching ongoing mental operations (Bruning et al., 2020, 2021, 2022).

An important aspect of theorizing concerns the effect of practice on test reliability, which is crucial in situations where repeated testing takes place (Hagemeister, 2007; Hagemeister and Kronmaier, 2017). According to Logan (1988), the most general way of theorizing on practice effects is to assume two distinct processes of solving speeded arithmetic, a calculation-based process and a process based on memory retrieval. Critical is that both processes are running in parallel and in competition to each other. Performance is considered automatic when based on single-act, direct-route retrieval of results from memory, while it is considered controlled when based on algorithmic processing such as counting, adding, memorizing, borrowing (Ashcraft, 1992), or negating a logical term (Deutsch et al., 2006, 2009; Goodwin and Johnson-Laird, 2011, 2013). Key to this idea is that these processes race against each other so that each unique trial is finally cleared up by either the retrieval or the algorithmic operation. Practice gains (due to retesting) occur, according to this conception, because repeated exposure leads to an accumulation of separate episodic traces with experience, which gives them a race advantage over the algorithmic process (Miller and Ulrich, 2003, 2013; Han and Proctor, 2022a,b,c). To say it another way, retesting produces a gradual transition from algorithmic processing to memory-based processing and thus changes the relation (i.e., the mixture parameter) of both as a function of amount of practice (Compton and Logan, 1991; Pashler and Baylis, 1991a,b; Steinborn et al., 2009, 2010b; Los et al., 2014, 2017, 2021; Crowe and Kent, 2019).

Although the concept of coordination, which lies at the core of the mental demands in Düker-style tasks, is considered a trait-like characteristic of normal individuals (Düker, 1949; Bruning et al., 2021), it is also widely resorted to in clinical and neuropsychological contexts to assess the level of cognitive functioning in patients (Bates and Lemay, 2004; Stuss et al., 2005), or wherever individual-case assessment in this cognitive domain is indicated (Willmes, 1985; Stuss et al., 2001). In a broader sense, coordination is a natural ingredient of many everyday activities and of high relevance to research on every-day multitasking (Botvinick and Bylsma, 2005a,b; Salvucci and Taatgen, 2008, 2011). It is crucial to the understanding of individual differences in cognitive performance (Ackerman, 1987; Ackerman and Kanfer, 2009; Steinborn et al., 2016, 2018; Bruning et al., 2020, 2022), though any strict definition naturally depends on the particular (task- and time-based) characteristics (Thomaschke et al., 2012; Thomaschke and Dreisbach, 2015). A study of Bruning et al. (2021) performed an in-depth analysis of individual differences and found a great diversity of how a task is represented by individuals and how this determines response organization (cf. Phillips and Rabbitt, 1995; Watson and Strayer, 2010; Cheyne et al., 2011; Steinborn et al., 2012; Schumann et al., 2022).

### 1.2. Multitrait-multimethod approach

A unique feature of both elementary and complex (integrated multiple-act) speed tests is that the they are administered in a self-paced mode, which places particular emphasis on the supervisory monitoring of the proper speed–accuracy balance (cf., Rabbitt and Banerji, 1989; Jentzsch and Leuthold, 2006), and that several ways of measuring performance are possible, each with a distinct meaning (Steinborn et al., 2016, 2018). While average performance speed is typically considered the primary measure, error rate serves as a secondary measure, held to indicate rigor, diligence, or punctiliousness, or a lack thereof, respectively (Bates and Lemay, 2004). Speed and accuracy are often not or only modestly correlated which is taken as an argument for the discriminant validity of both measures (Flehmig et al., 2007; Steinborn et al., 2016, 2018; Blotenberg and Schmidt-Atzert, 2019a,b). It is often ignored, however, that error scores (with errors being rare events) exhibit a skewed population distribution, which limits test reliability, which again poses a limit on correlational relationships with other performance indices. Test guidelines often recommend combining measures of speed and accuracy into a single compound dimension, either as penalty-based combination score (Bruyer and Brysbaert, 2011; Gropel et al., 2014; Steinborn et al., 2016, 2018; Wuhr and Ansorge, 2020), or on grounds of model-based reasoning (Vandierendonck, 2017; Liesefeld and Janczyk, 2019), others resort to throughput, defined as the rate of work in a given time frame (Thorne, 2006; Szalma and Teo, 2012). In self-paced tasks, combining speed and accuracy to a compound measure of (inversed) efficiency often yields a slight improvement of reliability (Pieters, 1983, 1985; Van Breukelen et al., 1995; Steinborn et al., 2018, p. 350).

Recent research increasingly focused on measuring the fluctuation of performance, and connected with this point, how this concept could be indexed reliably (Jensen, 1992; Leth-Steensen et al., 2000; Flehmig et al., 2007; Unsworth, 2015; Steinborn et al., 2016, 2018; Fortenbaugh et al., 2017; Unsworth and Robison, 2020). A majority of studies examining intra-individual performance variability resorted to reaction-time standard deviation (RTSD), which seems natural at first sight, given that most statistics textbooks refer to *SD* as the appropriate measure of dispersion of metric-scale data points around their arithmetic mean. However, RTSD is, for pure mathematical reasons, highly correlated with the mean response time (RTM), and due to this redundancy, of only limited diagnostic value. Flehmig et al. (2007) suggested the response time coefficient of variation (RTCV) to index variability, which relates RTSD to the individual’s RTM, yielding an index of variability relative to the individual’s overall level of performance speed (cf. Steinborn et al., 2016, 2018). RTCV is calculated by dividing the individual RTSD by the individual RTM, multiplied by 100: RTCV = (RTSD/RTM) × 100. As a result, a measure is obtained that allows for comparing intra-individual RT variability beyond mere–scaling variability (Wagenmakers and Brown, 2007; Steinborn et al., 2017) and is thus suitable for comparing variability even of individuals who differ very much in their average cognitive speed (Jensen and Rohwer, 1966; Neubauer and Fink, 2009).

Miller and Ulrich (2013) have recently developed the individual-differences in response time (IDRT) model which is based on classical test theory and introduces the analysis of standard psychometric criteria (i.e., reliability, convergent vs. discriminant validity, stability) within an RT modeling framework (cf. Campbell and Fiske, 1959; Steinborn et al., 2016, 2018). This approach enables a formal and systematic investigation of the question of which aspects of mental processing time affect the reliabilities and correlations of RT-based measures as assessed with standard psychometric tests. According to the IDRT model, empirically observed RTM is composed of three separate components, individual differences in global processing speed (cf. Bruning et al., 2021; Bruning et al., 2022, for a theoretical view), processing time that is imposed by a certain experimental variable (e.g., effect of increasing workload), a residual term, and an error term. Briefly, IDRT can be described as being composed of (1) person-specific general processing time, (2) task-specific processing time, (3) residual time, and (4) measurement error. In this regard, Miller and Ulrich’s theorizing implicates a hierarchical evaluative analysis of empirical correlations within both measures of same test’s performance and across measures of different tests’ performance (Cronbach and Meehl, 1955; Campbell and Fiske, 1959; Gleser et al., 1965; Rajaratnam et al., 1965; Steinborn et al., 2016, 2018; Hedge et al., 2018).

The *first* precondition is to evaluate whether the population parameter of all performance scores exhibit a sufficient level of symmetry and variance (see Table 1). A skewed population distribution would indicate lack of variance either because of a bottom or a ceiling effect (Dunlap et al., 1994; Greer et al., 2006). If there is no information in the data, there will be no expectation of a potential correlation. For example, error scores in speeded tests often reveal a skewed distribution and thus lack test reliability thereof (Hagemeister, 2007; Steinborn et al., 2012; Wessel, 2018). In the *second* step, the reliability diagonal is evaluated which is the precondition to obtain correlations with other measures. This is particularly important in studies where measurable entities are claimed as being *“independent”* concepts. The *third* step is to determine convergent validity, indicating the degree to which a theoretically related concept is actually interrelated empirically. The *fourth* step is to determine discriminant validity, indicating the degree to which a theoretically unrelated concept is not interrelated empirically. In order to determine construct validity of the target measures, one has to demonstrate reliability as a precondition and both convergence and discrimination. Mental arithmetic is especially suitable for constructing tests because of its flexibility to generate items with desirable psychometric characteristics. This concerns two aspects, finding adequate levels of difficulty (e.g., varying problem size) and mitigating practice gains from repeated testing (e.g., increasing item set). Using staggered multi-step processing by combining multiple mental operations (e.g., addition combined with subsequent verification, negation, comparison, memorization) allows for a flexible arrangement of items enabling various options to control for psychometric criteria (Restle, 1970; Ashcraft, 1992; Rickard, 2005; Steinborn et al., 2012).

**TABLE 1:** A brief guide to understanding the logic underlying the Multitrait-multimethod (MTMM) approach to individual differences in multitasking.

	Step	Metaphor and symbolic assumptions
**1**	Skew check	- the *molecular* precondition: symmetry of population distribution - skew indicates lack of variance through bias (bottom or ceiling effect) - skew → no information in the data → no expectation of correlation - example: rare events (errors, oddball effects, etc.) & self-ratings often skewed
**2**	Reliability	- the *molar* precondition: reliability limits correlational relationships between variables - typical retest intervals: 1–2 weeks for performance tests, 4 weeks for questionnaires - factors affecting reliability: number of trials, scaling differences (metric vs. %) - required for claim such as, e.g., “…*results show that the constructs are independent*…”
**3**	Convergence	- theoretically similar concepts → expected to be related empirically - is given when two indicators representing the same concept are highly correlated - e.g.: “ability” convergently represented through indicators (efficiency vs. throughput) - a way of judging indicator utility
**4**	Discriminance	- theoretically different concepts → expected not to be related empirically - is given when two indicators representing different concepts are not correlated - e.g., a high correlation of RTM with RTSD could be seen as lack of discrimination - often produced by method similarity, or natural mathematical relations
**5**	Stability	- concerns the degree to which (absolute) scores remain constant from test to retest - stability → true score (ability) of person has not changed after repeated testing - main factors biasing stability: test-taker strategies and practice gains - practice gains often unequal across participants → impede reliability scores
**6**	Reproducibility	- concerns the between-session correlational structure - comparison of correlation structure above vs. below the MTMM reliability diagonal - indicates that the convergent and divergent relationships are stable and reproducible - in essence, a qualitative way of judging the robustness of a nomological network
**7**	Generalizability	- concerns the replicability of the overall findings with conceptually similar tests - judging whether conclusions are specific or can be made with some scope of validity - generality of findings generates essence → scientific substance → knowledge - on principle, a serial process of corroborating scope and substantiality of concepts

The points 1–2 are basic preconditions that must be fulfilled in order to enable any expectation about relationships of variables with each other. The points 3–4 are the classic evaluation dimensions of the MTMM, as they give an indication of how close (vs. distant) concepts are empirically. The points 5–7 are, in a strict sense, qualitative dimensions of credibility control, achieved through a serial process of replication with slightly varied conceptual variation (cf. Stroebe and Strack, 2014, pp. 61–63; Miller and Ulrich, 2021, 2022).

### 1.3. Analysis of integrated Multiple–Act arithmetic

Here, we asked whether common performance measures obtained from integrated multi-act arithmetic exhibit sufficient test–retest reliability, discriminant validity, and robustness against practice from repeated testing. We followed the reasoning implied by the IDRT model (Miller and Ulrich, 2013) and the multitrait–multimethod approach (Campbell and Fiske, 1959; Steinborn et al., 2018) as a heuristic means to evaluate convergent and discriminant validity of competing performance measures. To this end, we investigated test–retest reliability, correlational structure, and liability to practice effects, indexing average speed (RTM), error percentage (EP), absolute (RTSD), and relativized (RTCV) reaction-time variability. Data of two experiments are presented, each requiring the integrated coordination of elemental acts. In Experiment 1, we analyzed performance using a mental addition and verification paradigm (Zbrodoff and Logan, 1986, 1990; Steinborn and Huestegge, 2016, 2017, 2020), where participants are presented with an addition term including its result (e.g., 2 + 3 = 5), and are required to verify the correctness of the term by pressing a “yes” or “no” response. In Experiment 2, we used an integrated dual-act mental addition and comparison paradigm (Restle, 1970), where individuals are presented with an addition problem and with a single number, which are spatially separated by a vertical line (e.g., “4 + 5 | 10”); they are instructed to solve the addition problem and then to compare the number value of their calculated result with the number value of the presented digit.

## 2. Experiment 1

### Methods

#### Participants

Twenty-nine young adults (age between 20–30, recruited at the Dresden University of Technology) participated in the study. It is intrinsic to the study of test reliability to have a diversified sample population with a (relatively) balanced gender ratio, including participants not only from the faculty of psychology but also from other faculties (the humanities, natural sciences, etc.). There were two testing sessions with a retest interval of 1 week, which took place under similar conditions (i.e., at the same place and at about the same time). Participants had normal or corrected-to-normal vision and reported to be in normal health condition.

#### Material and apparatus

**FIGURE 1:**
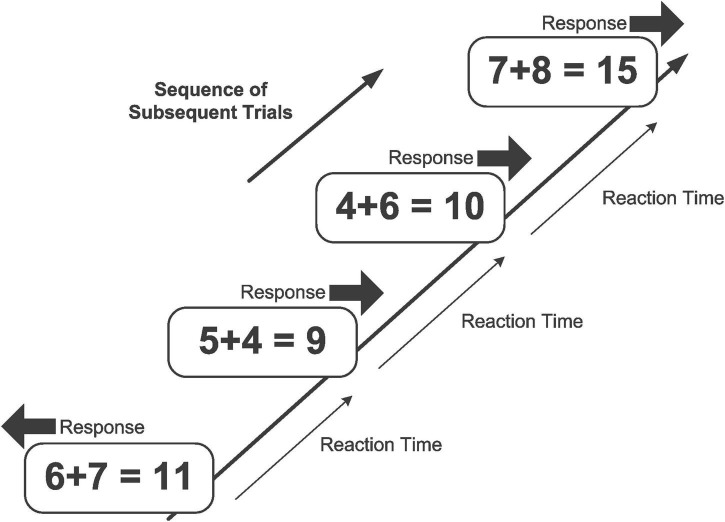
Example of a series of trials in the mental addition and verification task. Participants have to perform an addition operation (e.g., “3 + 4 =”) and then to verify (or falsify) the correctness of their mental result with the presented addition term (e.g., “7 = 7”), by pressing the right (or left) response key. The task is self-paced such that each item is presented immediately after the response to the previous item.

The self-paced mental addition and verification was administered twice within a retest interval of 3 days (cf. Figure 1). Each item in a trial was presented until response, and was replaced immediately after the response (RSI = 50 ms) by the next item (Steinborn and Huestegge, 2016, 2017, 2020). No feedback was given, neither in case of an erroneous response, nor in case of too slow responses. In a typical trial, they were presented with an addition problem including its result, either correct or incorrect (e.g., “4 + 5 = 9”, or “4 + 3 = 6”). Problem size was varied to a maximum of 19 (i.e., 2 + 3; 3 + 2 …8 + 7, 7 + 8), and ties (e.g., 2 + 2, 3 + 3, 4 + 4, …, etc.) were excluded (cf. Blankenberger, 2001). Participants were required to solve the addition problem and then to decide whether the result is correct or incorrect. In case of a correct result (in half of all trials), they had to press the right button (“yes”), and in case of an incorrect result, they had to press the left button (“no”). In an experimental session, 428 trials were presented. The task required no more than about 30 min of testing time.

#### Procedure and design

The experiment took place in a noise shielded room and was run on a standard personal computer with color display (19”, 100 Hz frequency), controlled *via* the software Experimental Runtime System (ERTS), developed by Behringer (1987). Participants were seated at a distance of about 60 cm in front of the computer screen, and the stimuli were presented at the center of the screen.

### Results and discussion

Population parameters and correlations are displayed in Tables 2, 3. The first five responses were regarded warming-up trials and not considered for analysis. Responses faster than 100 ms were regarded outliers and discarded from response time analysis. Correct reactions within this interval were used to compute averaged response time (RTM), standard deviation of response times (RTSD), and coefficient of variation of response times (RTCV). Incorrect responses were computed to index error percentage (EP). MTMM analysis served to evaluate reliability as well as convergent and discriminant correlations of the alternative performance indices in the self-paced speed tests.

**TABLE 2:** Descriptive statistics for Experiment 1 and Experiment 2.

Experiment 1 (serial mental addition and verification task)									
		Session 1				Session 2			
			
	Measures	M	SD	Skew	Range	M	SD	Skew	Range
1	RTM	1402	333	0.23	848–2042	1270	297	0.50	774–2028
2	RTMc	1460	359	0.15	866–2113	1322	304	0.48	794–2112
3	ER	3.86	2.78	0.96	0.23–11.19	3.94	3.68	2.34	0.47–18.18
4	RTSD	679	318	0.25	189–1390	618	280	0.06	185–1141
5	RTCV	0.46	0.14	0.14	0.21–0.78	0.47	0.15	–0.11	0.23–0.76

**Experiment 2 (serial mental addition and comparison task)**									

		**Session 1**				**Session 2**			
			
	**Measures**	**M**	**SD**	**Skew**	**Range**	**M**	**SD**	**Skew**	**Range**

1	RTM	2063	471	0.78	1191–3035	1744	391	0.51	196–2744
2	RTMc	2128	481	0.78	1237–3229	1785	396	0.52	1133–2776
3	ER	3.05	2.59	3.35	0.33–16.97	2.34	1.97	1.34	0.16–9.14
4	RTSD	1074	494	1.25	383–2743	809	316	0.44	290–1.708
5	RTCV	0.50	0.14	1.59	0.30–1.03	0.45	0.10	0.10	0.26–0.72

Population parameters for all performance measures. *N* = 29 (Exp. 1); *N* = 50 (Exp. 2); RTM, response time mean; RTMc, error-corrected RTM (inversed efficiency); ER, error percentage; RTSD, response time standard deviation; RTCV, response time coefficient of variation.

**TABLE 3:** Multitrait-multimethod-matrix for Experiment 1 and Experiment 2.

Experiment 1 (serial mental addition and verification task)						
		Session 1				
		
Session 2		1	2	3	4	5
RTM	*1*	0.93**	0.99**	0.05	0.89**	0.69**
RTMc	*2*	0.99**	0.94**	0.18	0.89**	0.70**
ER	*3*	–0.17	–0.01	0.84**	0.13	0.22
RTSD	*4*	0.88**	0.88**	–0.04	0.91**	0.93**
RTCV	*5*	0.59**	0.62**	–0.07	0.90**	0.91**

**Experiment 2 (serial mental addition and comparison task)**						

		**Session 1**				
		
**Session 2**		**1**	**2**	**3**	**4**	**5**

RTM	1	0.94**	0.99**	–0.13	0.85**	0.63**
RTMc	2	0.99**	0.94**	–0.01	0.84**	0.62**
ER	3	–0.17	–0.08	0.78**	–0.11	–0.05
RTSD	4	0.88**	0.89**	–0.08	0.83**	0.93**
RTCV	5	0.59**	0.60**	0.05	0.89**	0.78**

Test–retest reliability and intercorrelation structure (convergent vs. divergent) of all performance measures, separately for session 1 and session 2. *N* = 29 (Exp. 1); *N* = 50 (Exp. 2); RTM, response time mean; ER, error percentage; RTSD, response time standard deviation; RTCV, response time coefficient of variation. Test–retest reliability is shown in the main diagonal (denoted with gray); correlations for the first session are shown above, for the second session below the main diagonal. **p < 0.01.

#### Retest reliability

Reliability coefficients are shown along the main diagonal of the correlation matrix (Table 3), presenting the correlations between the first and the second test administration. As expected, RTM was highly reliable (*r* = 0.93). Surprisingly, good reliability was also obtained for ER (*r* = 0.84), given that error-score reliability is for the most task low or insufficient (Maloney et al., 2010; Steinborn et al., 2016, 2018; Hedge et al., 2018, for an overview). Note that error scores are often reported as not sufficiently reliable, due to the fact that errors are rare events in chronometric tasks (Jentzsch and Dudschig, 2009; Notebaert et al., 2009; Steinborn et al., 2012; Steinhauser et al., 2017; Wessel, 2018; Dignath et al., 2020; Schaaf et al., 2022), particularly in self-paced psychometric tests (Hagemeister, 2007). Most interestingly, not only RTSD but also RTCV appeared to be highly reliable indices of performance (*r* > 0.91), a finding that deviates a bit from previous studies that demonstrated insufficient reliability for the relativized response speed variability measures.

#### Correlational structure

There was no relationship between RTM and ER indicating discriminant validity (Table 3). Pronounced (expected) relationships were found between absolute and relativized variability as represented by RTSD and RTM (*r* = 0.89 and *r* = 0.87). In fact, the relationship shows that both measures represent a similar aspect of performance. Substantial positive correlations were also found between RTCV and RTM, albeit to a lesser degree (*r* = 0.69 and *r* = 0.60). This is in contrast to the previously reported findings where RTCV was observed as less reliable and uncorrelated to RTM.

#### Practice effects

We performed a repeated-measures analysis of variance including practice (test vs. retest) as factor and performance as dependent measures. A multivariate effect was observed from the first to the second testing session for all performance measures. A more detailed analysis of separate effects revealed that only RTM and RTSD were significantly affected by test–retest practice gains. As expected, individuals became faster on average after practice, indicated by the effects of session on RTM [10% gain; *F*(1,28) = 35.1; *p* < 0.000; η*^2^* = 0.56] and on RTSD [10% gain; *F*(1,28) = 6.3; *p* < 0.05; η^2^ = 0.19].

#### Extended analyses

Miller and Ulrich (2013, p. 824) argued that the average response speed (RTM) should strongly be influenced the intraindividual variability of the same test, as reflected by RTCV, so reliability should decrease as CV increases. The authors argue that “…*naturally, researchers should take steps to minimize trial-to-trial fluctuations in arousal, attention, and other factors that might increase RT variability*….” Since this assumption remains largely untested for the case of test–retest variability, we here examined it. To this end, we compared groups of individuals who exhibited high versus low performance in the self-paced task (as a proxy to simulate patient populations), partitioning the sample into two groups of individuals according to their RTM, so that each group contained 50% of the sample. Note that though the model theorizes on individual differences in variability, most people would naturally expect to divide them according to speed as critical dimension, with average speed partly depending on variability. For simplicity, therefore, we decided to use speed instead of variability, but we state here the use of variability reveals a very similar pattern. We observed that the reliability of RTM was largest for the high-performance group (*r* = 0.98^**^) but was considerably decreased for the low-performance group (*r* = 0.81^**^). This finding shows that even with an enormous amount of trials per experimental session (i.e., 424 trials in Exp. 1), RTM measures became less reliable when the analysis was restricted to a subgroup of individuals exhibiting performance deficits, as typically observed in patient groups (Stuss et al., 1996, 2005).

## 3. Experiment 2

### Method

#### Participants

The sample comprised 50 participants (70% female) who were recruited *via* advertisements and at the Campus of the Dresden University of Technology. All participants had normal or corrected-to-normal vision, and all of them reported to be in good health.

#### Material and apparatus

**FIGURE 2:**
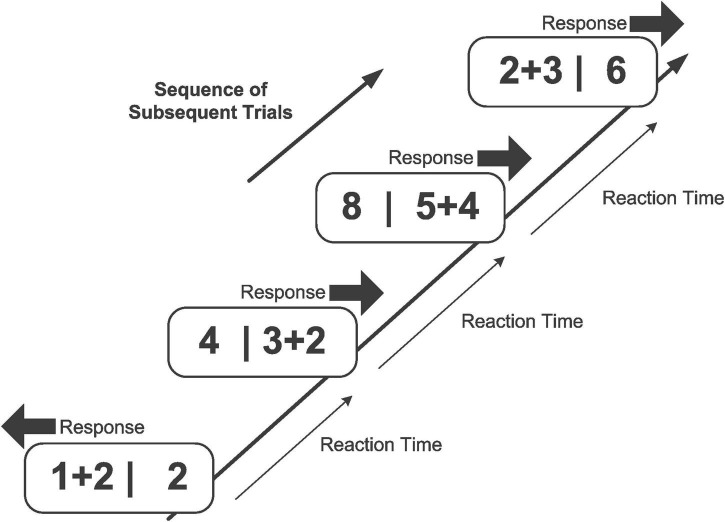
Example of a series of trials in the mental addition and comparison task. The task is to solve the addition term (e.g., “2 + 3 = … ?”) and to decide whether the result is larger or smaller than the presented number value (e.g., “5 > 6 … ?”). The participants are to respond by “choosing” the larger of the number values. Each item in the task is presented immediately after the response to the previous item.

The serial mental addition and comparison test (Restle, 1970) was administered twice within a retest interval of 4 days (cf. Figure 2). Participants self-paced their responding since each item in a trial was presented until response, replaced “immediately” (i.e., 50 ms RSI) after responding by the next item. No feedback was given, neither in case of an erroneous response, nor in case of too slow responses. In a typical trial, they were presented with an addition problem and with a single number, spatially separated by a vertical line (e.g., “4 + 5 | 10”). They were to solve the addition problem and to compare the number value of their calculated result with the number value of the presented digit. In all trials, the value of the digit was either one point smaller or one point larger than the value of the addition term but never of equal value. Participants were required to “choose” the larger number value by pressing either the left or the right key. That is, when the number value on the left side was larger (e.g., “2 + 3 | 4”), they had to respond with the left key, and when the number value on the right side was larger (e.g., “5 | 2 + 4”), they had to respond with the right key. Thus, the task required coordinated addition and comparison demands. The item set contained 148 items, with ties being excluded (cf. Blankenberger, 2001), and with a problem size ranging from 4 to 19. Both the large number of items (set-size effect) and the diversification of item difficulty (*“peek-a-boo”* uncertainty effect) are effective means to preventing mindless (rhythmic) responding (Lupker et al., 1997; Schmidt et al., 2016; Braem et al., 2019; Schumann et al., 2022), similar to the shuffling of preparatory intervals (Grosjean et al., 2001; Steinborn et al., 2009, 2010b; Langner et al., 2010, 2011, 2018; Wehrman and Sowman, 2019, 2021). In a session, each item was presented four times, amounting to a total of 592 randomly presented trials, requiring no longer than 30 min of testing.

### Results and discussion

Population parameters and correlations are displayed in Tables 2, 3. Responses faster than 100 ms were discarded, correct reactions within this interval were used to compute RTM, RTSD, and RTCV. Incorrect reactions were regarded as error.

#### Retest reliability

Response time was again highly reliable (*r* = 0.94), and also good reliability was obtained for ER (*r* = 0.79) and RTSD (*r* = 0.83). Most important, both indices of performance variability appeared to have good reliability (*r* = 0.83 for RTSD; *r* = 0.78 for RTCV).

#### Correlational structure

Response time and ER were uncorrelated. Strong correlations were found between RTSD and RTM (*r* > 0.85 and *r* = 0.88), corroborating the redundancy of both measures. On the other hand, substantial positive correlations were also found between RTCV and RTM (*r* > 0.63 and *r* = 0.59), again indicating that even mean-corrected variability has some conceptual overlap with speed.

#### Practice effects

A multivariate effect was revealed overall and separately for all measures. A more detailed analysis revealed substantial practice gains for RTM [18% gain; *F*(1,49) = 169.7; *p* < 0.000; partial η^2^ = 0.78], RTSD [32% gain; *F*(1,49) = 41.6; *p* < 0.000; partial η^2^ = 0.46], and ER [27% gain; *F*(1,49) = 9.9; *p* < 0.001; partial η^2^ = 0.17]; less pronounced gains were observed for RTCV [11% gain; *F*(1,49) = 16.5; *p* < 0.000; partial η^2^ = 0.25]. Thus, participants became faster, more accurate, and more constant after retesting.

#### Extended analyses

**FIGURE 3:**
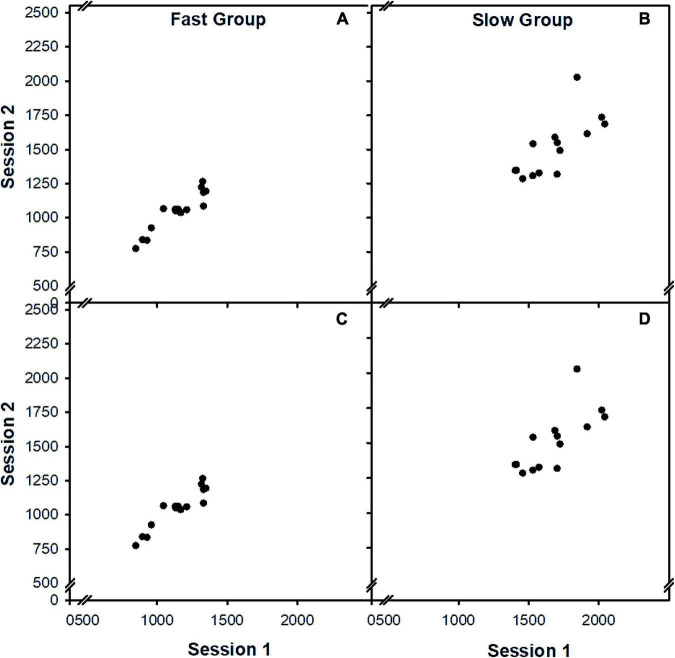
Results of Experiment 1 and Experiment 2. Scatterplot of the relationship between test and retest performance for both Experiment 1 (Panels **A,B**) and 2 (Panels **C,D**), separately for a group of fast individual and a group of slow individuals.

We again and in the same way compared low- vs. high-performance participants. Reliability of RTM was largest for the low-performance group (*r* = 0.92) but was decreased for the high-performance group (*r* = 0.79), indicating again that even with many trials per experimental session (i.e., 529 trials in Exp. 1), RTM measures became prone to unreliability in slightly deficient subgroups (cf. Figure 3).

## 4. General discussion

The main results can be summarized as follows: (1) *Molecular precondition:* There was an approximately symmetric (sample-population) distribution (except skew in error rate), permitting to expect correlations between and across sessions. (2) *Molar precondition* (*reliability)*: The relevant performance indices exhibited high test–retest reliability as obtained from the correlation of two testing sessions. This utility effect arguably stems from two sources, the time-compression property (Miller and Ulrich, 2003, 2013) and multitasking property (Vandierendonck et al., 2010; Vandierendonck, 2017). (3–4) *Convergence and discriminance*: Speed (RTM) and error rate (ER) were uncorrelated, both within and across testing sessions, indicating discriminant relationships. Response speed variability as indexed by RTCV was both reliable and relatively uncorrelated to other performance dimensions such as speed and accuracy, qualifying RTCV as discriminant from measures of speed and accuracy. By contrast, absolute RT variability (RTSD) was highly correlated with RTM, indicating redundancy. (5) *Stability:* Further, there were substantial practice gains for RTM and RTSD, and partly for ER; however, RTCV remained relatively stable after retesting – a finding that is consistent with the previous result of Flehmig et al. (2007). (6) *Reproducibility*. The correlational structure was relatively similar at the first relative to the second testing session, indicating stable relationships. (7) *Generalizability*. The overall picture of reliability, correlational structure and stability was similar in both tasks, enabling similar overall conclusions, and supporting the claim that speed tests based on multi-act mental arithmetic are promising in the experimental study of mental testing.

### 4.1. Reliability, inter-correlation, practice effects

#### Reliability

In order to construct a test with excellent psychometric characteristics, one has to consider three aspects, the *principles of measurement theory* (Lord and Novick, 1968; Lienert, 1969) the principles of *chronometric-design theory* (Miller and Ulrich, 2013, 2021, 2022), and the principles of *concept-generalizability theory* (Cronbach and Meehl, 1955; Stroebe and Strack, 2014, pp. 61–63). On the side of measurement theory, the reliability of a test is determined by the amount of true score variance relative to error variance, and this ratio increases with number of trials. On the side of design theory, the amount of processing time should be maximal relative to testing time (Schumann et al., 2022, pp. 14–15). To this end, we employed a sufficient trial number in a self-paced presentation mode. As a result, exceptional test–retest reliability was obtained for the speed measure (*r* = 0.90). With respect to error rate (ER), retest reliability was surprisingly good (*r* = 0.79), given that error rate is typically low in self-paced tasks (∼5%), which limits reliability of error scores for pure mathematical reasons. However, low-event rate unreliability can partially be compensated by aggregating the absolute number of errors through lengthening a test (Hagemeister, 2007), that is, by trading off test economy with test reliability (Steinborn et al., 2016, 2018). Remarkable is that RTCV as relativized measure of variability exhibited good reliability in both tasks, given the many reports of a lack of reliability. How can these findings be accounted for? According to Miller and Ulrich (2013), reliability is predicted to increase with individual differences in person-specific processing time and individual differences evoked by task demands, but to decrease with increasing residual time and measurement error. Likely, exceptional reliability was obtained because mental arithmetic enables diversification of individual items: it is possible to use a great number of different individual items, preventing practice effects, and it allows for a finely graduated variegation of item difficulty (e.g., problem size ranging from 3–19), which mitigates mindlessness (Sanabria et al., 2011; Bedi et al., 2022) or other kinds of unfocused rhythmic responding (Steinborn and Langner, 2011, 2012; Schmidt, 2017; Braem et al., 2019).

#### Convergence and discriminance

Campbell and Fiske (1959) provided a practical methodology for purposes of construct validation and test construction. At its heart are two types of validity termed convergent and discriminant, defined as subcategories of construct validity. Convergent validity refers to the degree to which concepts that are related theoretically are actually interrelated empirically (Gleser et al., 1965; Rajaratnam et al., 1965). Discriminant validity refers to the degree to which theoretically distinct concepts are not interrelated empirically. To determine construct validity of a test’s target measure is to demonstrate convergence and discrimination. Across experiments, speed and error rate were uncorrelated, indicating discriminant validity. RTCV was uncorrelated to error rate but somewhat related to speed, yet to a lesser degree than RTSD. Given the high reliability, this would clearly qualify RTCV over RTSD as the measure of choice to index performance variability. *One could ask, at this point, why a correlation of RTCV with RTM is still evident in a relativized (mean-corrected) measure like RTCV*? According to Flehmig et al. (2007), the answer is that while RTSD shares natural commonality with RTM for pure mathematical reasons, a relativized (percentage-based) measure captures performance variability beyond mere scaling–variability (Wagenmakers and Brown, 2007; Steinborn et al., 2016, 2018). A relationship between RTM and RTCV is still possible when an experimental factor evokes variability through propagation, or when variability is evoked along the trait (i.e., ability) dimension, for example, in a situation where lower ability is *in a specific way* related to more distraction during a task. A final note: we strongly advise against the method of removing shared variance of RTM with RTSD by means of partial correlation, as is sometimes seen in the literature, for two reasons. First, the obtained “mean-corrected” residual of a variability is “sample-dependent,” that is, it cannot be interpreted individually (e.g., each individual added to a sample would change the score values for all other individuals of the sample). In general, we would not recommend indices computed from z-standardized scores, either as sum, subtractive, or residual score, or based on covariance-analytical techniques, as found in the literature (e.g., Seli et al., 2013; Klein and Robinson, 2019; Liesefeld and Janczyk, 2019), because they are unsuitable in practical assessment situation, or in clinical diagnostics (Willmes, 1985; Stuss, 2006; Vallesi and Shallice, 2007).

#### Practice effects

In theory, a test should be robust against practice effects. In reality, however, this requirement is unrealizable, since virtually every thinkable psychometric test will be affected by retesting, at least to some extent (Lemay et al., 2004; Calamia et al., 2013; Scharfen et al., 2018a,b,c; Soveri et al., 2018; Blotenberg and Schmidt-Atzert, 2019a,b; Williams et al., 2022). Crucial to the evaluation of a test is whether retesting changes the psychometric properties, which is relevant in situations where retesting is unavoidable, such as in the study of sleep deprivation, memory, or some sort of patient studies (Bratzke et al., 2009, 2012; Strobach et al., 2012; Mattiesing et al., 2017; Kunchulia et al., 2022). The results of the present two experiments demonstrate that despite retesting yielding substantial practice gains, the interrelation of the performance measures remained stable throughout. Overall, we can consider convergent and discriminant validity of the performance measures as being similar at both testing sessions, and across both experiments. Most interesting, the relativized measure of performance variability (RTCV) was to a much lesser degree affected by practice as compared to the measure of central tendency (RTM), which demonstrates that the relative performance fluctuations that occur during the test are stable with repeated testing. From a psychometric perspective, this is a remarkable feature of RTCV of assessing performance effects in psychometric speed tests (Pieters, 1983, 1985; Steinborn and Huestegge, 2016, 2017, 2020).

### 4.2. Multitasking measures in cognitive psychometrics

The study of multitasking is both multifarious and multitudinous given the multiple definitions and paradigms proposed in the literature. Depending on the particular objective, research can be classified into three categories, one concerned with the cognitive analysis of tasks (Pashler, 1994a,b; Meyer and Kieras, 1997a,b; Hommel, 1998a,b; Salvucci and Taatgen, 2008, 2011), another with the “human” factor at work and leisure (Wickens, 2008; Parasuraman and Manzey, 2010; Schumann et al., 2022) as well as in competitive sports activities (e.g., Kunde et al., 2011; Wehrman and Sowman, 2019, 2021; Pedraza-Ramirez et al., 2020; Polzien et al., 2022), and the third one with the utility of cognitive operations for psychometric test construction (Miller and Ulrich, 2003, 2013; Steinborn et al., 2016, 2018). By definition, multitasking can be conceived of as a special form of performance behavior that requires more than one mental act at a time or in close succession with the unit of observation being either a manifest or latent variable. For example, in a dual-response paradigm (Huestegge and Koch, 2013; Raettig and Huestegge, 2021), the performance registration is directly observable, while in a multi-act (chained) arithmetic paradigm, the units of interest lay hidden and can only indirectly be inferred (Pieters, 1983, 1985). Since even the simplest decision imposes a considerable demand requiring multiple inhibitory control of alternative response options (Moeller and Frings, 2019; Raettig and Huestegge, 2021), it is utterly impossible to reach a definition of multitasking that is minimalistic and universal at the same time (Ackerman, 1987; Logan, 2002, 2004; Altmann and Gray, 2008; Kiesel et al., 2010; Paas Oliveros et al., 2022; Schumann et al., 2022).

#### Düker-type vs. Bourdon-type tests

In the psychometric discipline (Jensen and Rohwer, 1966; Jensen, 1992; Miller and Ulrich, 2003, 2013), cognitive theory serves the practical purpose of constructing tests that meet standard psychometric criteria, and this division is thus more open to the general definition with respect to mental operations. According to Düker, capacity is *created through coordination of elemental acts*, which can be conceived of as the well-ordered orchestration of internal operations in close temporal proximity toward completing the ongoing task (Groen and Parkman, 1972; Ashcraft, 1992; Huber et al., 2016). The speeded arithmetic of the present study fall into this category of tasks. In the mental addition and comparison test (Exp. 1), for example, an addition problem is presented together with a single number, both spatially separated (e.g., “4 + 5 | 10”). Completing the task requires two elemental acts, solving the addition problem, and comparing the result with the number value of the presented digit (Restle, 1970; Steinborn et al., 2012). In this way, Düker-type tasks represent a natural form of an integrated-dual task, and by this means, provide opportunities of studying (sub-)task integration processes in goal-oriented multitasking (Restle, 1970; Treisman and Gelade, 1980; Huber et al., 2016; Moeller and Frings, 2019; Pedraza-Ramirez et al., 2020). With this regard, the use of culture techniques such as adding or subtracting numbers or multitasking combinations in gamified environments (Bilalic et al., 2009; Meyerhoff et al., 2017; Strobach and Huestegge, 2017; Pedraza-Ramirez et al., 2020) are superior as task medium for psychometric testing (Restle, 1970; Ashcraft, 1992; Huber et al., 2016), both in paper-pencil and computerized testing (Steinborn et al., 2016, 2018; Wuhr and Ansorge, 2020), in laboratory and real-assessment situations (Schumann et al., 2022).

#### Guidelines for constructing speed tests

According to Cronbach (1975), there are five reasons why the use of more complex speed tests based on mental addition are preferable: (a) test reliability, (b) test economy, (c) culture fairness, (d) flexibility, (e) and broadband validity (Thorndike, 1971; Jensen, 1980; Wuhr and Ansorge, 2020). In general terms, speed tests deliver a measure to assess ability as broadband concept, typically indicated by a wide spectrum of criterion validity. The basic principle is to employ a large number of trials and to administering the test in a self-paced mode so that the processing of items are compressed per unit of time, capturing maximal processing time relative to overall testing time (Miller and Ulrich, 2003, 2013). While 5–10 min of testing time seems optimal, it should not exceed 20–30 min. Although it is not exactly clear how complex a task should be to reach optimal reliabilities, medium task complexity (RT’s approximately 1–2 s, 5–10% errors) seems to be a good option, as indicated by the present results and previous findings (cf. Schumann et al., 2022, for a review). Increasing complexity beyond some point (RTs > 3 s) is “increasingly” problematic as error rate will dramatically escalate, which produces ambiguity on the speed–error relation, for both (easy vs. hard) task condition and (slow vs. fast) individuals. Setting optimal levels and variegating (shuffling) difficulty are the most important control options for test development, typically achieved by varying arithmetic problem size (Ashcraft, 1992; Imbo and Vandierendonck, 2008a,b), arithmetic-chain length (Steinborn and Huestegge, 2016, 2017, 2020), or by using forms of multi-act mental arithmetic.

#### Indexing performance variability

Across two experiments, we observed both RTSD and RTCV as being sufficiently reliable with respect to retesting, indicating that this measure does not reflect random fluctuations but systematic variance that is replicable at a second testing session. Therefore, the correlations of measures of central tendency and variability may be interpreted without being concerned about insufficient reliability (Miller and Ulrich, 2013), as sometimes argued by a “reliability paradox” (Hedge et al., 2018). In fact, the correlations were around *r* = 0.60 at both first and second testing session and in both experiments (Table 3), indicating discriminant validity. Since the correlation of RT and RTSD were much higher, being between values of *r* = 0.85–0.89, the present results show that RTCV is the measures that should be preferred when one intends to measure performance variability in practical assessment contexts. This feature makes RTCV quite interesting for practical assessment purposes where test validity is at danger of being compromised by prior test experience (Hagemeister, 2007). If a measure is significantly affected by retesting, and an individual’s performance level before practice cannot be determined, it becomes impossible to separate potential practice effects from the individual’s ability, which the test was constructed to measure (Cronbach, 1975, p. 310–312). Failure to use appropriate control techniques would then lead to erroneous inferences about the aptitude of the individual being tested. Of course, further research is needed to examine whether invariance to practice effects is a general property of RTCV or only specific to a certain category of tasks. According to our findings, RTCV might be a potential candidate for characterizing additional aspects of performance in a simple and efficient way, in both basic research and applied contexts. However, before applying RTCV in practical assessment settings, additional research is required to elucidate the impact of task-specific factors (i.e., optimizing item difficulty, item-set size, task length, etc.) on the reliability of this performance measure using the MTMM approach.

### 4.3. Conclusion

Among the various assessment instruments available, speed tests based on cultural techniques (e.g., coding, sorting, arithmetic) typically exhibit the highest degree of test reliability (Neubauer and Knorr, 1998; Stahl and Rammsayer, 2007; Steinborn et al., 2010a; Wuhr and Ansorge, 2020). In terms of psychometric characteristics, speed tests stand any comparison to virtually all popular test batteries aiming to assess executive functions with typical testing times of 60–90 min (Zimmermann and Fimm, 1993; Fan et al., 2002; Westhoff and Graubner, 2003; Habekost et al., 2014). Self-paced tests are also frequently used to experimentally induce a state termed ego depletion (Hagger et al., 2010; Inzlicht and Schmeichel, 2012; Krishna and Strack, 2017; Inzlicht et al., 2018; Vohs et al., 2021), albeit there are substantial methodological weaknesses of many reported studies in this area, relating to aspects like the use of arbitrary tasks, unsound performance measures, or an insufficient number of trials. Some studies even concluded that ego depletion is particularly measurable in the variability of performance rather than in RT mean score (Massar et al., 2018; Satterfield et al., 2018; Kamza et al., 2019; Unsworth and Robison, 2020). The reason might lie in the nature of depletion phenomena. Given that it is not the specific process but the control of attention, that is, the superordinate process of adaptively regulating mental resources, one would predict that performance becomes unstable rather than simply slow. The key contribution of the present psychometric analysis therefore covers at least three aspects, knowledge and step-by-step guidance in terms of how to correctly analyze and evaluate the psychometric properties of multitasking measures, methodology of design and research logic within the framework of mental chronometry using multi-act mental arithmetic, and proper (simple but robust) measurement technology. The central message of our report is that individual-differences in multitasking as assessed *via* correlations are not interpretable by themselves but must be evaluated in the light of their *molecular* and *molar* preconditions that involve, according to our analysis, at least 7 *formal steps of evaluation* (Table 1) to be worked through one after another.

## Data availability statement

The raw data supporting the conclusions of this article will be made available by the authors, without undue reservation.

## Ethics statement

Ethical review and approval was not required for the study on human participants in accordance with the local legislation and institutional requirements. The patients/participants provided their written informed consent to participate in this study.

## Author contributions

FS, MS, and HF: concept and method. JK, RL, and LH: supervision and discussion. All authors contributed to the article and approved the submitted version.
